# High Frequency of Fusion Transcripts Involving *TCF7L2* in Colorectal Cancer: Novel Fusion Partner and Splice Variants

**DOI:** 10.1371/journal.pone.0091264

**Published:** 2014-03-07

**Authors:** Torfinn Nome, Andreas M. Hoff, Anne Cathrine Bakken, Torleiv O. Rognum, Arild Nesbakken, Rolf I. Skotheim

**Affiliations:** 1 Department of Cancer Prevention, Institute for Cancer Research, Norwegian Radium Hospital - Oslo University Hospital, Oslo, Norway; 2 Centre for Cancer Biomedicine, Faculty of Medicine, University of Oslo, Oslo, Norway; 3 University of Oslo, Oslo, Norway; 4 Department of forensic pathology and clinical forensic medicine, Division for forensic medicine, The Norwegian Institute of Public Health, Oslo, Norway; 5 Department of Gastrointestinal Surgery, Oslo University Hospital-Aker, Oslo, Norway; University of Illinois at Chicago, United States of America

## Abstract

*VTI1A-TCF7L2* was reported as a recurrent fusion gene in colorectal cancer (CRC), found to be expressed in three out of 97 primary cancers, and one cell line, NCI-H508, where a genomic deletion joins the two genes [1]. To investigate this fusion further, we analyzed high-throughput DNA and RNA sequencing data from seven CRC cell lines, and identified the gene *RP11-57H14.3* (ENSG00000225292) as a novel fusion partner for *TCF7L2*. The fusion was discovered from both genome and transcriptome data in the HCT116 cell line. By triplicate nested RT-PCR, we tested both the novel fusion transcript and *VTI1A-TCF7L2* for expression in a series of 106 CRC tissues, 21 CRC cell lines, 14 normal colonic mucosa, and 20 normal tissues from miscellaneous anatomical sites. Altogether, 42% and 45% of the CRC samples expressed *VTI1A-TCF7L2* and *TCF7L2-RP11-57H14.3* fusion transcripts, respectively. The fusion transcripts were both seen in 29% of the normal colonic mucosa samples, and in 25% and 75% of the tested normal tissues from other organs, revealing that the *TCF7L2* fusion transcripts are neither specific to cancer nor to the colon and rectum. Seven different splice variants were detected for the *VTI1A-TCF7L2* fusion, of which three are novel. Four different splice variants were detected for the *TCF7L2-RP11-57H14.3* fusion. In conclusion, we have identified novel variants of *VTI1A-TCF7L2* fusion transcripts, including a novel fusion partner gene, *RP11-57H14.*3, and demonstrated detectable levels in a large fraction of CRC samples, as well as in normal colonic mucosa and other tissue types. We suggest that the fusion transcripts observed in a high frequency of samples are transcription induced chimeras that are expressed at low levels in most samples. The similar fusion transcripts induced by genomic rearrangements observed in individual cancer cell lines may yet have oncogenic potential as suggested in the original study by Bass et al.

## Introduction

Colorectal cancer (CRC) is the second most common and deadly cancer disease world-wide [2], and there is high demand of good biomarkers for both early detection and to stratify patients according to prognosis and predicted treatment responses. However, CRC is a heterogeneous disease, and few biomarkers have yet made it to routine clinical use.

Fusion genes represent one class of cancer genes with promising biomarker potential, and when caused by chromosomal rearrangements such as translocations, deletions or duplications, they are commonly highly cancer specific. So far, few highly recurrent fusion genes have been identified in CRC. Examples from other cancer types includes the well-known *BCR-ABL1* fusion (Philadelphia chromosome), present in 95% of chronic myelogenous leukemias [3], and *TMPRSS2-ERG* which is present in around 50% of prostate cancers [4]. In some cases, the fusion gene can be a therapeutic target, demonstrated by Imatinib binding to and blocking the kinase domain of the BCR-ABL1 fusion protein.

The first fusion gene reported to be recurrent in CRC, fusing sequences of *VTI1A* and *TCF7L2*, was reported in 2011 [1]. *VTI1A-TCF7L2* fusion transcripts were detected in three out of 97 (3%) CRCs, as well as the colon cancer cell line NCI-H508. In NCI-H508, the fusion is caused by a ∼540 kb deletion between the genes *VTI1A* and *TCF7L2*. *TCF7L2* encodes the TCF4 transcription factor, which dimerizes with β-catenin. β-catenin is involved in regulation of the WNT-signaling pathway, which is commonly altered in the majority, if not all, CRCs [5]. Depletion of the *VTI1A-TCF7L2* fusion transcript resulted in significant loss of anchorage independent growth in the fusion positive CRC cell line NCI-H508 [1].

Frequent mutations in a polyadenine tract within the *TCF7L2* gene have previously been reported for CRC, especially in microsatellite instable tumors [6]. Microsatellite stable tumors have, however, also recently been shown to harbor frequent mutations in the *TCF7L2* gene, indicating a possible important function in CRC biology [7].

In this study, we aimed to investigate further the presence and variants of *VTI1A*-*TCF7L2* fusions in CRC. We have analyzed deep sequencing whole-genome and transcriptome data from CRC for related fusions involving one of the fusion partners, and for novel fusion breakpoints. The recurrence of the *VTI1A-TCF7L2* fusion, and a new fusion transcript between *TCF7L2* and the novel partner gene *RP11-57H14.3,* was analyzed in a series of CRCs and normal samples.

## Materials and Methods

### Material

A total of 106 CRC tissue samples and 14 paired normal samples from two independent patient series, Series 1 and Series 2, were screened for presence of the fusion transcripts. All CRCs are from patients treated surgically at hospitals in the Oslo region, Norway. The two patient series included both microsatellite stable (n = 85) and instable (n = 20) CRCs (one sample not scored), as assessed by previous studies [8]–[10]. The series were enriched for clinical stages II and III CRC (52 stage II, 53 stage III, and 1 stage IV). Staging was in accordance to the American Joint Cancer Committee/Union for International Cancer Control (AJCC/UICC). The 14 paired normal samples from Series 1 were collected from visually disease free areas of colonic mucosa. The research biobanks have been registered according to national legislation, and the study has been approved by the Regional Committee for Medical Research Ethics (numbers 2781 and 236-2005-16141).

A total of 21 colorectal cell lines were included [11]. The cell lines HCT116, HCT15, LoVo, NCI-H508, RKO, SW48, and SW620 were purchased from the American Type Culture Collection (ATCC, Manassas, VA, USA). The cell lines Co115, Colo320, EB, FRI, HT29, IS1, IS2, IS3, LS1034, LS174T, SW480, TC7, TC71, and V9P were kindly provided by Dr. Richard Hamelin, Inserm, France. Identities of the cell lines were verified by the AmpFLSTR Identifiler PCR Amplification Kit (Applied Biosystems by Life Technologies, Carlsbad, CA, USA).

Additionally, we screened twenty normal tissues from miscellaneous sites of the body for the *TCF7L2* involving fusion transcripts (adipose, bladder, brain, cervix, colon, esophagus, heart, kidney, liver, lung, ovary, placenta, prostate, skeletal muscle, spleen, stomach, testes, thymus, thyroid and trachea; FirstChoice Human Normal Tissue Total RNA, each a pool of RNA from at least three individuals, with the exception of an individual sample from the stomach; Ambion, Applied Biosystems by Life Technologies, Carlsbad, CA, USA).

### Identification of Fusions from Whole-transcriptome and Whole-genome Sequencing Data

Paired-end RNA-sequencing data from seven colorectal cancer cell lines (HCT15, HCT116, HT29, LS1034, RKO, SW48, and SW480) and from 16 normal tissues of miscellaneous origins were included in the study. These were all sequenced using the Illumina GAIIx (cell lines) or HiSeq 2000 (normal tissues; both sequencers from Illumina Inc., San Diego, CA, USA). The colon cancer RNA-seq data included 220 million 76 bp sequence reads (European Nucleotide Archive study accession ID ERP002049) [12] and the normal samples included between 73 and 80 million 50 bp sequence reads per sample (ArrayExpress accession id [E-MTAB-513] and European Nucleotide Archive study [EMBL:ERP000546]). Additionally, we obtained paired-end whole-genome sequences of the cell lines HCT15, HCT116, HT29 and SW480 to an average coverage of about 30×[12] (Data may be made available upon request to researchers).

A list of potential fusion transcripts was produced by deFuse version 0.6.0 [13] on the seven previously mentioned RNA-sequenced cell lines in addition to the CRC cell line NCI-H508 (analysis id 0c7a79cc-bbaf-4c6d-93e1-866f5f5f3d0d) downloaded from Cancer Genomics Hub (hosted by the University of California Santa Cruz, CA, USA), data provided by the Cancer Cell Line Encyclopedia [14] and 16 tissues from the Illumina Human Body Map v2. For the cell lines with paired whole-genome sequence (HCT116, HCT15, HT29 and SW480), RNA fusion transcripts and DNA breakpoints were identified by nFuse version 0.2.0 [15]. A fusion nomination required three spanning read pairs and two split reads from the RNA-seq data.

### Detection of Fusion Transcripts by Reverse-transcriptase PCR

For the *VTI1A-TCF7L2* fusion, the same RT-PCR primers and nested primers were used as in the original publication [1]. For the *TCF7L2-RP11-57H14.3* fusion, primers and nested primers were designed to the fusion transcript breakpoint sequence, as identified by deFuse, by utilizing the Primer3 web software [16]. The optimal primer sequences (Table S1) were further ordered from BioNordika Norway AS (Oslo, Norway) and synthesized by Eurogentec (Liège, Belgium). We performed sensitive nested RT-PCR with 20+30 cycles for both assays using 50 ng of cDNA from each sample as input into first round PCR. The products of the first PCR were diluted to a final concentration of 1/200 in the nested PCR. The following cycling protocol was used for all PCR reactions: 15 minutes of HotStarTaq DNA polymerase activation at 95°C, a three-step cycle of denaturation for 30 seconds at 95°C, primer annealing for one minute and 15 seconds at optimal primer melting temperatures (Table S1), and extension for one minute at 72°C. After the last cycle, a final extension step was performed at 72°C for 6 minutes. The nested-PCR products were separated by electrophoresis at 200 V for 30 minutes on a 2% agarose gel and visualized using ethidium bromide and UV light. Triplicate RT-PCR runs were performed for both assays, using identical parameters, for all samples. No template negative controls were included from each cDNA synthesis, first-round PCR and nested-PCR reactions.

### Sanger Sequencing of Fusion Transcript Breakpoints

Samples that were positive for the second replicate RT-PCR assays, and showed a single nucleotide band on the agarose gel, were sequenced directly from both sides using both forward and reverse nested primers. Prior to sequencing, the nested PCR products were purified using Illustra ExoStar 1-step cleanup (GE Healthcare, Little Chalfront, UK). The cycle sequencing reactions were performed using the BigDye Terminator v.3.1 cycle sequencing kit (Applied Biosystems, Foster City, CA, USA) following supplier’s recommendation. Further, the sequencing products were cleaned and purified using BigDye Xterminator (Applied Biosystems), before they were analyzed by capillary electrophoresis using the ABI 3730 DNA Analyzer (Applied Biosystems). The sequences were analyzed using the Sequencing Analysis v.5.3.1 software.

### Assessment of the Identified Genomic Breakpoint *TCF7L2-RP11-57H14.3* by PCR

The genomic breakpoint identified from whole-genome sequencing data, connecting *TCF7L2* to *RP11-57H14.3* in the colon cancer cell line HCT116, was validated using PCR.

Primers for genomic PCR (Table S1) were designed to the genomic breakpoint sequence, as identified by nFuse, using the same approach as described above for the RT-PCR assays. Twenty-one CRC cell lines, including HCT116, were tested for the identified genomic breakpoint using 100 ng of DNA as input for each reaction. The following cycling protocol was used for the genomic PCR: 15 minutes of HotStarTaq DNA polymerase activation at 95°C, a three-step cycle (repeated 35 times) of denaturation for 30 seconds at 95°C, primer annealing for one minute and 15 seconds at 60°C and extension for one minute at 72°C. After the last cycle, a final extension step was performed at 72°C for 6 minutes. The PCR products were separated by electrophoresis at 200 V for 30 minutes on a 2% agarose gel, and visualized using ethidium bromide and UV light.

## Results

### *RP11-57H14.3* is a Novel Fusion Partner in *TCF7L2* Containing Fusion Transcripts

**Figure 1:**
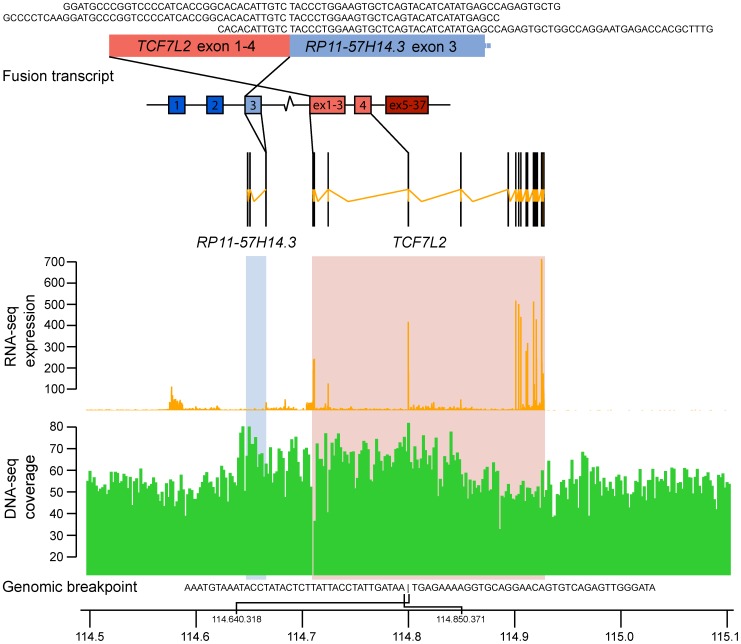
The *TCF7L2-RP11-57H14.3* fusion transcript, identified in the HCT116 cell line, harbors a rearranged genomic locus. Three chimeric RNA sequence-reads spanned the fusion transcript breakpoint, passing from exon 4 of *TCF7L2* (ENST00000369395) to exon 3 of *RP11-57H14.3* (ENST00000428766), on chromosome 10. Dark colors indicate exons not part of the fusion transcript. RNA-seq expression and DNA-seq coverage levels are based on sequencing data of the HCT116 cell line. The two gene loci are marked in blue and red boxes. The genomic breakpoint sequence as identified by nFUSE in the CRC cell line HCT116 is given; spanning from the intronic region of *TCF7L2* to upstream of *RP11-57H14.3*. The coordinates of the breakpoint (chr10∶114,850,371-114,640,318 (GRCh37)) are marked on the chromosome position axis. The location of the breakpoint correlates well with the increased genomic coverage seen from the genome sequencing data.

To search for the *VTI1A-TCF7L2* fusion and novel fusion partners, we analyzed paired-end RNA-sequencing data from eight colon cancer cell lines and sixteen normal tissue samples from miscellaneous anatomical sites. By applying the software deFuse, we identified between 16 and 1050 potential fusion transcripts per sample. From whole genome sequencing data of four colon cancer cell lines, the nFuse software identified between 70 and 126 potential genomic breakpoints. Of all fusions identified, only NCI-H508 harbored the *VTI1A-TCF7L2* fusion transcript, with a single breakpoint spanning from exon two in *VTI1A* to exon six in *TCF7L2*, as already reported for this cell line by Bass et al. [1]. However, we identified a genomic breakpoint (chr10∶114,850,371-114,640,318 (GRCh37)) in the CRC cell line HCT116 that spanned from the intronic region of *TCF7L2* to upstream of *RP11-57H14.3* (ENSG00000225292) with a corresponding RNA fusion (Table 1). *RP11-57H14.3* is located in the intergenic region between *VTI1A* and *TCF7L2* approximately 44 kb upstream of *TCF7L2*. The predicted genomic breakpoints correlate with increased genomic coverage in the region between them, suggesting a genomic duplication causing the fusion (Figure 1). Both the genomic breakpoint and fusion transcript between *TCF7L2* and *RP11-57H14.3* were verified by PCR and RT-PCR. The identified genomic breakpoint was verified in the HCT116 cell line, but not detected in any of the 20 other cell lines tested, thereby reducing the likelihood that the genomic breakpoint reflects a common DNA copy number polymorphism.

**Table 1:** nFuse and defuse: Verification of the original *VTI1A-TCF7L2* fusion transcript and identification of a fusion transcript and genomic breakpoint involving *TCF7L2* and *RP11-57H14.3.*

Cell line	GeneA	GeneB	Software	Split^†^	Spanning^†^	Score
HCT116	*RP11-57H14.3*	*TCF7L2*	deFuse	3	4	0,84^a^
			nFuse	NA	27	5,5^b^
NCI-H508	*VTI1A*	*TCF7L2*	deFuse	64	27	0,93^a^

*RP11-57H14.3* is located 44 kb upstream of *TCF7L2* on chromosome 10.

a)deFuse probability score,

b)nFuse path score. †) Split reads contain the fusion boundary in the read itself, while spanning reads are paired ends that harbor the fusion boundary within the insert sequence.

### High Prevalence of *TCF7L2*-involving Fusion Transcripts, Both Involving *VTI1A* and the *RP11-57H14.3* Genes

**Figure 2:**
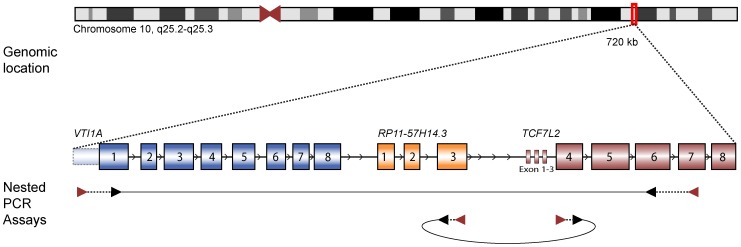
Schematic presentation of the genomic location of *VTI1A, RP11-57H14.3* and *TCF7L2*. All three genes are located within 720*VTI1A,* ENST00000428766 in *RP11-57H14.3* and ENST00000369395 in *TCF7L2*. Also, the nested PCR assays used for detection of both fusion transcripts are shown. The red and black arrows represent the first round and second round primers used, respectively.

Using the same primers as described by Bass et al. [1] (Figure 2), we detected *VTI1A-TCF7L2* fusion transcripts with different exon-exon combinations in 45 out of 106 CRC samples (42%) (Table 2). Fusion transcripts were as well detected from 4 of 14 normal colonic mucosa samples. Out of the 14 paired tumor-normal samples, eight pairs were positive exclusively in tumor, three pairs were positive in both tumor and normal, and one pair positive exclusively in normal (Table 3). Prevalence of fusion transcripts was similar in microsatellite stable and instable tumors. Ten out of 21 cell lines, including the NCI-H508, harbored fusion transcripts of different sizes. Finally, five normal tissue samples from different anatomical sites of the body expressed the fusion transcripts (Table S2). Because the RT-PCR results revealed inconsistent results (Figure S1 and Figure S2), we performed all RT-PCRs in triplicate, with identical parameters, to investigate recurrence of fusion transcripts (Table S3). Fusion transcripts were only detected consistently in all three runs in four samples; three tumor samples and the NCI-H508 cell line (3.1% of all CRC samples and cell lines). This frequency is similar to the originally identified frequency of *VTI1A-TCF7L2* transcripts (3%) by Bass et al. [1].

**Table 2:** The number of positive fusion transcript PCRs for the samples, run in triplicates.

	n =	Strong Positives^a^:*VTI1A-TCF7L2*	Positives^b^:*VTI1A-TCF7L2*	Strong Positives^a^:*TCF7L2-RP11-57H14.3*	Positives^b^:*TCF7L2-RP11-57H14.3*
Series 1-tumor	14	1 (7.1%)	11 (79%)	4 (29%)	9 (64%)
Series 1-normal	14	0	4 (29%)	0	4 (29%)
Series 2	92	2 (2.2%)	34 (37%)	2 (2.2%)	39 (42%)
Cell lines	21	1 (4.8%)	11 (52%)	9 (43%)	19 (90%)
Normals	20	0	5 (25%)	5 (25%)	15 (75%)

a)Strong positives are defined as testing positive in all RT-PCR replicates.

b)Samples noted as positives have tested positive for the fusion transcript(s) in one or more of the three RT-PCR replicates.

**Table 3:** Matched tumor and normal colonic mucosa from series 1.

	*VTI1A-TCF7L2*		*TCF7L2-RP11-57H14.3*	
Pair #	Tumor	Normal	Tumor	Normal
1	Y	Y	N	N
2	Y	N	N	N
3	Y	N	Y	Y
4	Y	N	Y	N
5	Y	N	Y	Y
6	Y	N	Y	N
7	N	N	N	N
8	N	N	Y	N
9	Y	Y	Y	Y
10	N	Y	Y	Y
11	Y	N	N	N
12	Y	N	N	N
13	Y	N	Y	N
14	Y	Y	Y	N

For both fusion transcripts the tumor, or both the tumor and normal samples were frequently positive. In one pair the *VTI1A-TCF7L2* fusion transcript was positive only in the normal sample and not the matched tumor.

We detected *TCF7L2-RP11-57H14.3* fusion transcripts with different exon-exon combinations in 48 out of 106 CRC samples (45%; Table 2). Out of the 14 paired tumor-normal samples, five pairs were positive exclusively in tumor, and four pairs were positive in both tumor and normal (Table 3). 19 out of 21 cell lines, harbored fusion transcripts of different sizes. Also, 15 out of 20 normal tissue samples were positive for fusion transcripts (Table S2). As with the *VTI1A-TCF7L2* fusion, we performed all RT-PCRs in triplicate to investigate the recurrence of fusion transcripts (Table S3). In total there were 20 samples that repeatedly tested positive for fusion transcripts; six tumor samples, five samples from normal tissues and nine cell lines (including the HCT116 cell line). Interestingly, the NCI-H508 cell line was negative for *TCF7L2-RP11-57H14.3* fusion transcripts in all three replicates.

We found no correlation between CRCs positive for *TCF7L2* containing fusion transcripts involving *VTI1A* and *RP11-57H14.3* (p = 0.33; Fisher’s Exact test). Further, the frequencies of fusion transcripts were not significantly different between microsatellite instable *vs.* stable tumors, nor between clinical stages (data not shown).

All nested-PCR replicates for both assays contained negative no template controls. These negative control reactions never produced detectable PCR-products (Figure S1 and S2).

### Chimeric Sequences Generally Covered Intact Exonic Splice Sites from the Partner Genes

**Figure 3:**
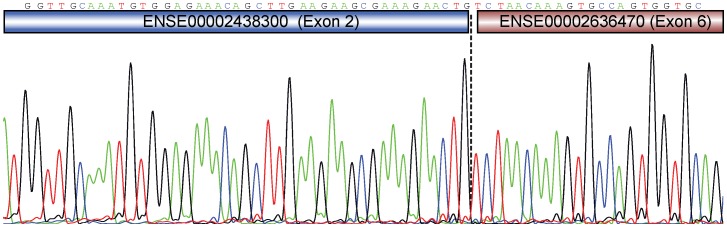
Confirmation of the original *VTI1A-TCF7L2* fusion transcript breakpoint in the cell line NCI-H508. Sanger sequencing confirmed the original fusion transcript discovered in NCI-H508, showing the breakpoint sequence spanning exon-exon junctions between exon 2 in *VTI1A* and exon 6 in *TCF7L2*. Bass et al. also discovered three other fusion transcripts by nested-PCR. However, as transcript annotation was not sufficiently described, we are not able to say if these fusions are identical to some of the transcripts we have identified.

From one of the RT-PCR runs, all samples that were positive for the fusions and had a single PCR product were selected for Sanger-sequencing of the chimeric RNA-sequences to identify the exact breakpoints. For *VTI1A-TCF7L2* (n = 25), we obtained sequences from all 25 such isolated fusion transcript RT-PCR products, where 24 out of 25 had sequences connecting upstream sequences of the exons 1, 2, 3, 5 or 7 of *VTI1A* to downstream sequences of the exons 4 or 6 of *TCF7L2*, with preservation of the same exon-exon boundaries as in the already annotated gene structures (ENST00000393077 in *VTI1A* and ENST00000369395 in *TCF7L2*). One sequenced product did not contain clear exon-exon boundaries, but connected the two transcripts in middle of exon 7 in *VTI1A* and 6 in *TCF7L2*. In total, seven different fusion breakpoints were identified (Table S3) including the original breakpoint between exon 2 of *VTI1A* and exon 6 of *TCF7L2* in NCI-H508 (Figure 3) [1]. This exact breakpoint was not found in any of the other samples, but most of the intact fusion transcripts consisted of the same part of *TCF7L2* (n = 17) with some having exon 4 of *TCF7L2* spliced to exon 6 (n = 7). The *VTI1A* upstream contribution varied more, having five different combinations connecting to downstream *TCF7L2* parts.

For *TCF7L2-RP11-57H14.3* (n = 27), we obtained sequences from all 27 isolated fusion transcript RT-PCR products, where 26 out of 27 had sequences connecting sequences of exon 4 of *TCF7L2* to sequences of exons 1, 2, or 3 of *RP11-57H14.3*, also with preservation of the same exon-exon boundaries as in the already annotated gene structures (exon numbering is the same as above for *TCF7L2* and according to ENST00000428766 for *RP11-57H14.3*). One curious case of chimeric sequence, identified in the CRC cell line FRI, was a fusion transcript spanning three genes in a non-canonical genomic order, joining *TCF7L2* exon 4 with *VTI1A* exons 5, 6, and 7, and further extending into *RP11-57H14.3* exons 1, 2, and 3. Also in this unique case the same exon-exon boundaries were used as in the already annotated gene structures described above. In total, four different fusion transcripts were identified involving *TCF7L2-RP11-57H14.3,* all occurring in several samples each.

## Discussion

We have in the present report identified the gene *RP11-57H14.*3 as a novel fusion partner for *TCF7L2* in CRC. By sensitive nested RT-PCR, we have revealed that both the previously reported *VTI1A-TCF7L2* fusion transcripts, and the herein identified *TCF7L2-RP11-57H14.3*, are highly frequent among CRCs, although expressed at low levels. We also detected expression of the fusion transcripts in normal colonic mucosa, as well as in normal tissues from other anatomical sites. Triplicate nested-PCRs to investigate the presence of *TCF7L2* involving fusion transcripts showed variable results, with some samples initially testing positive for a fusion transcript, but negative when performing a consecutive run, or *vice-versa*. However, Sanger sequencing resulted in confirmation of clean exon to exon breakpoint junctions, reducing the likelihood that PCR artifacts, such as polymerase template switching [17], as a cause for the inconsistency. Negative controls were also performed at all RT-PCR steps, including cDNA synthesis, first-round PCR and nested-PCR. None of the negative controls resulted in detectable products, supporting that the high frequency of fusion transcripts observed is not a result of PCR contamination (Figure S1 and Figure S2). Although the fusion transcripts were found at high frequency within the tested biobank materials, the identification of *TCF7L2*-containing fusions from whole-transcriptome sequencing data was only successful from the two cell lines with matching genomic breakpoints. These two cell lines did as well have consistently strong expression of the fusion transcripts, as they produced consistent and clear RT-PCR results in all replicates. Based on these results, we suggest that the fusion transcripts produced between *TCF7L2* and either *VTI1A* or *RP11-57H14.3* are expressed at low levels in tumor samples, and some normal samples. The presence of fusion transcripts expressed at low levels are consistent with three fusion transcripts we recently identified in CRC, where *AKAP13-PDE8A, COMMD10-AP3S1,* and *CTB-35F21.1-PSD2* were identified in 17–58% of 106 primary cancer tissues [12].

All three partner genes are located on the long arm of chromosome 10, within 721 kbp, and are all read from the same strand in the order *VTI1A, RP11-57H14.3, TCF7L2* (Figure 2). This suggests RNA polymerase read-through as a potential mechanism for generating the *VTI1A-TCF7L2* transcripts in the absence of a corresponding genomic breakpoint. Several reports have shown that genes in close proximity in the human genome are expressed as conjoined genes, also called tandem chimeras, transcripts that are combined of at least part of one exon from two or more distinct genes that lie on the same chromosome [18]–[20]. It has been suggested that the expression of conjoined genes increase the complexity of the human genome by translating into distinct proteins, or that these transcripts play a role in regulation of canonical transcript levels. One suggested mechanism for their generation is that the transcription machinery avoids the termination signal of the upstream gene and continues transcribing the downstream gene before terminating at the downstream termination signal [19], [21]. The majority of the conjoined genes are believed to be expressed at low levels, as they are often supported by only a single expressed sequence tag or mRNA sequence in genome databases, which is in line with our observations of weakly expressed *TCF7L2*-containing fusion transcripts in CRC.

The generation of *VTI1A-TCF7L2* transcripts may well be explained as a product of polymerase read-through, but this cannot be the mechanism of operation for the *TCF7L2-RP11-57H14.3* fusion transcripts. In this case, the exons of *TCF7L2* and *RP11-57H14.3* are spliced together in a non-canonical genomic order using consensus splice-sites. This transcriptional mechanism has previously been described by Nigro et al. as exon scrambling; a process where exons are joined accurately at consensus splice sites, but in an order different from that present in the primary transcript [22]. They discovered this phenomenon when investigating a candidate tumor suppressor gene (*DCC*), and identified that the resulting scrambled transcripts are expressed and found at relatively low levels in both normal and neoplastic cells. Recently, based on deep sequencing of RNA, such scrambled transcripts from hundreds of genes were identified [23]. This group also suggested that a substantial fraction of the scrambled transcripts are circular RNAs, which explain the joining of exons in a non-canonical linear order. They also found that many of the circular isoforms were present at levels comparable to their canonical linear counterparts.

Altogether, these added levels of transcriptional and genomic complexity is in line with the recent report of the ENCODE project, reporting substantial reduction in the lengths of intergenic regions, and increasingly overlapping of genes previously assumed to be distinct genetic loci, altogether prompting a redefinition of the concept of a gene [24].

The identification of genomic breakpoints in individual cell lines for *TCF7L2* fusions both involving *VTI1A* and *RP11-57H14.3*, is intriguing. The genomic breakpoints identified coincide well with the frequently observed fusion transcripts discovered in other samples which do not have such genomic rearrangements. For the cell lines with identified genomic breakpoints, the fusion transcripts were detected at strong levels in all RT-PCR replicates, suggesting that these cells express the fusion transcripts at higher levels. Furthermore, the cell line with *VTI1A-TCF7L2* genomic fusion (NCI-H508) was negative for the *TCF7L2-RP11-57H14.3* fusion transcripts in all replicates, supporting the ∼540 kb genomic deletion of the intergenic region between *VTI1A* and *TCF7L2* originally identified by Bass et al.

The presence of the fusion transcripts in both normal cells and CRC samples together with identification of genomic breakpoints from individual cancer cell lines, joining the same two genes on the genome level, are in line with the report of the fusion transcript *JAZF1-JJAZ1* identified in both normal endometrial stromal cells and endometrial stromal tumors [25]. *JAZF1-JJAZ1* is detectable at the transcript level in normal endometrial stromal cells but genomic rearrangements are found only in the neoplastic cells. Li et al. hypothesize that trans-splicing generating these fusion transcripts, as well as other fusion transcripts, may occur regularly in normal cells and tissues. Further, they suggest that there may be a link between trans-splicing generating these fusion transcripts and the generation of the genomic rearrangements [26]. The genomic rearrangements or other mechanisms may lead to overexpression of these fusion transcripts, which may have oncogenic potential.

The importance of the gene *TCF7L2* in CRC development is favored by its function. *TCF7L2* encodes the TCF4 transcription factor, which is a key down-stream transcription factor in the WNT/β-catenin-signaling pathway, altered in the majority of CRCs [5]. The original report of *VTI1A-TCF7L2* found that depletion of the fusion transcript resulted in significant loss of anchorage independent growth in the fusion positive CRC cell line NCI-H508 [1]. Frequent mutations in a polyadenine tract within the *TCF7L2* gene have previously been reported for CRC, especially in microsatellite instable tumors [6]. Furthermore, microsatellite stable tumors have recently been shown to harbor frequent mutations in *TCF7L2* [7]. The observation that *TCF7L2* involving fusion transcripts are detectable in such a large fraction of CRC samples, but also normal colonic mucosa and other normal tissue types, reveals that the *TCF7L2* fusion transcripts are neither specific to cancer nor to the colon and rectum. Hence, they do not have the potential as cancer detection biomarkers as originally expected. When that is said, the similar fusion transcripts induced by genomic rearrangements observed in individual cancer cell lines may yet have oncogenic potential as suggested in the original study by Bass et al. The phenomenon of genomic rearrangements observed in cancer cells that correlate with transcription induced chimeras observed in most normal cells is in itself intriguing and needs to be explored further.

In conclusion, we have identified the gene *RP11-57H14.*3 as a novel fusion partner of *TCF7L2*. Both this fusion transcript and the previously reported *VTI1A-TCF7L2* are processed into several different splice variants, and *TCF7L2* involving fusion transcripts are expressed at detectable levels, in a high proportion of CRCs, and also in normal tissues from both colonic mucosa and from other anatomical sites. We suggest that these fusion transcripts are transcription induced chimeras, but that individual cancer cells have genomic rearrangements that lead to expression of highly similar fusion transcripts that potentially have a role in cancer development or progression.

## Supporting Information

Figure S1 ***VTI1A-TCF7L2*** **: Nested-PCR products in tumor samples, matched normals and CRC cell lines run in triplicate and analyzed on 2% agarose gels.** The results show a much higher degree of fusion-transcript positives than what has previously been reported for *VTI1A-TCF7L2*. However, the results diverge somewhat from run to run. A) Nested-PCR results from patient series 1 and 2. B) Nested-PCR results from the 21 CRC cell lines and some negative controls. The nested-PCR product of NCI-H508 seems more abundant compared to the other products based on the band luminescence. C) Additional negative controls, including no template controls from cDNA synthesis, first-round and second-round PCR.
(TIF)

Figure S2 ***TCF7L2-RP11-57H14.3*** **: Nested-PCR products in tumor samples, matched normals and CRC cell lines run in triplicate and analyzed on 2% agarose gels.** The results diverge somewhat for each replicate. A) Nested-PCR results from patient series 1 and 2. B) Nested-PCR results from the 21 CRC cell lines and some negative controls. There are at least three PCR-products from the cell line HCT116, which are present and identical in all replicates. C) Additional negative controls, including no template controls from cDNA synthesis, first-round and second-round PCR.
(TIF)

Table S1 **Primers used in this study.**
(DOCX)

Table S2 **Both fusion transcripts involving** ***TCF7L2*** **were detected, by RT-PCR, in several human** **tissues.**
(DOCX)

Table S3 **Total overview of nested RT-PCR and Sanger sequencing confirmation of fusion transcripts.**
(DOCX)
