# Supplementary figures and images for: High Frequency of Fusion Transcripts Involving TCF7L2 in Colorectal Cancer: Novel Fusion Partner and Splice Variants
Source: PLoS One. 2014 Mar 7;9(3):e91264. doi: 10.1371/journal.pone.0091264 (PMC3946716; doi:10.1371/journal.pone.0091264)

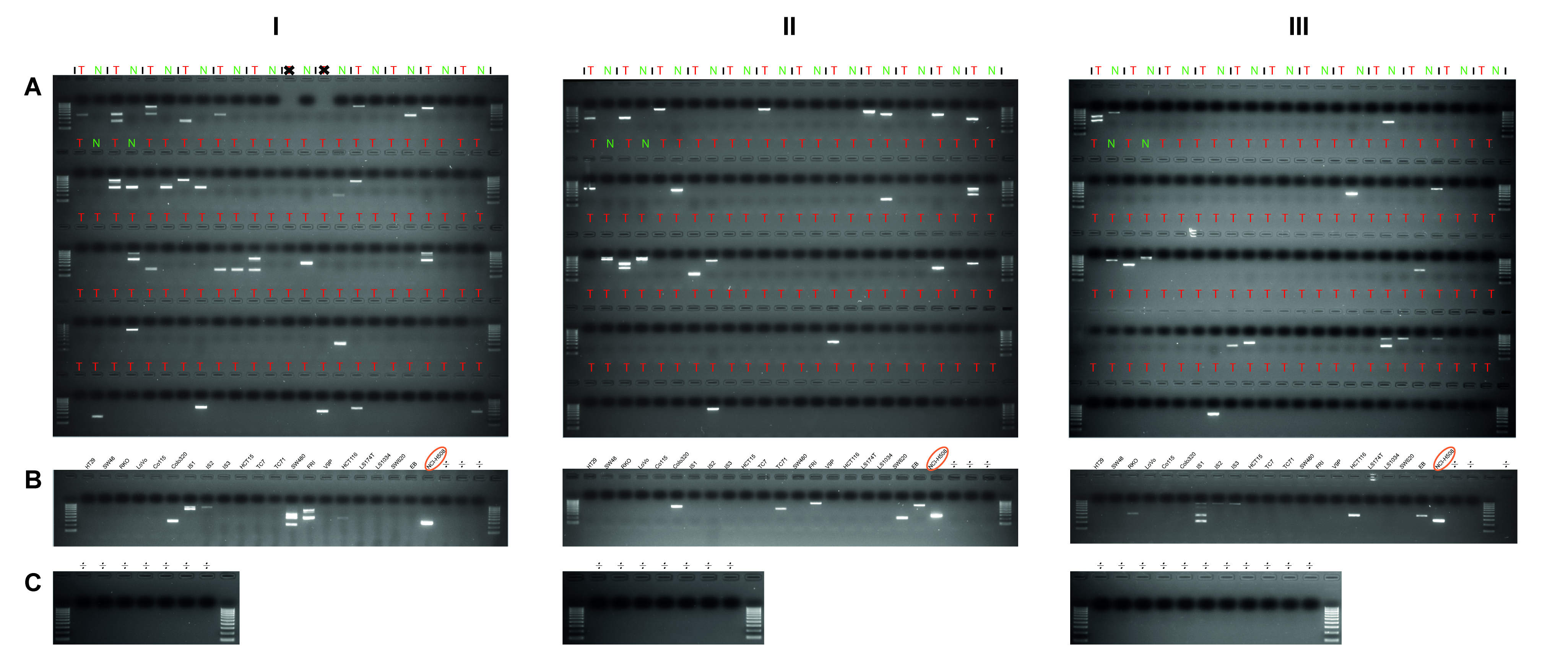

Supplement: Figure S1 — VTI1A-TCF7L2 : Nested-PCR products in tumor samples, matched normals and CRC cell lines run in triplicate and analyzed on 2% agarose gels. The results show a much higher degree of fusion-transcript positives than what has previously been reported for VTI1A-TCF7L2. However, the results diverge somewhat from run to run. A) Nested-PCR results from patient series 1 and 2. B) Nested-PCR results from the 21 CRC cell lines and some negative controls. The nested-PCR product of NCI-H508 seems more abundant compared to the other products based on the band luminescence. C) Additional negative controls, including no template controls from cDNA synthesis, first-round and second-round PCR. (TIF) [file pone.0091264.s001.tif]

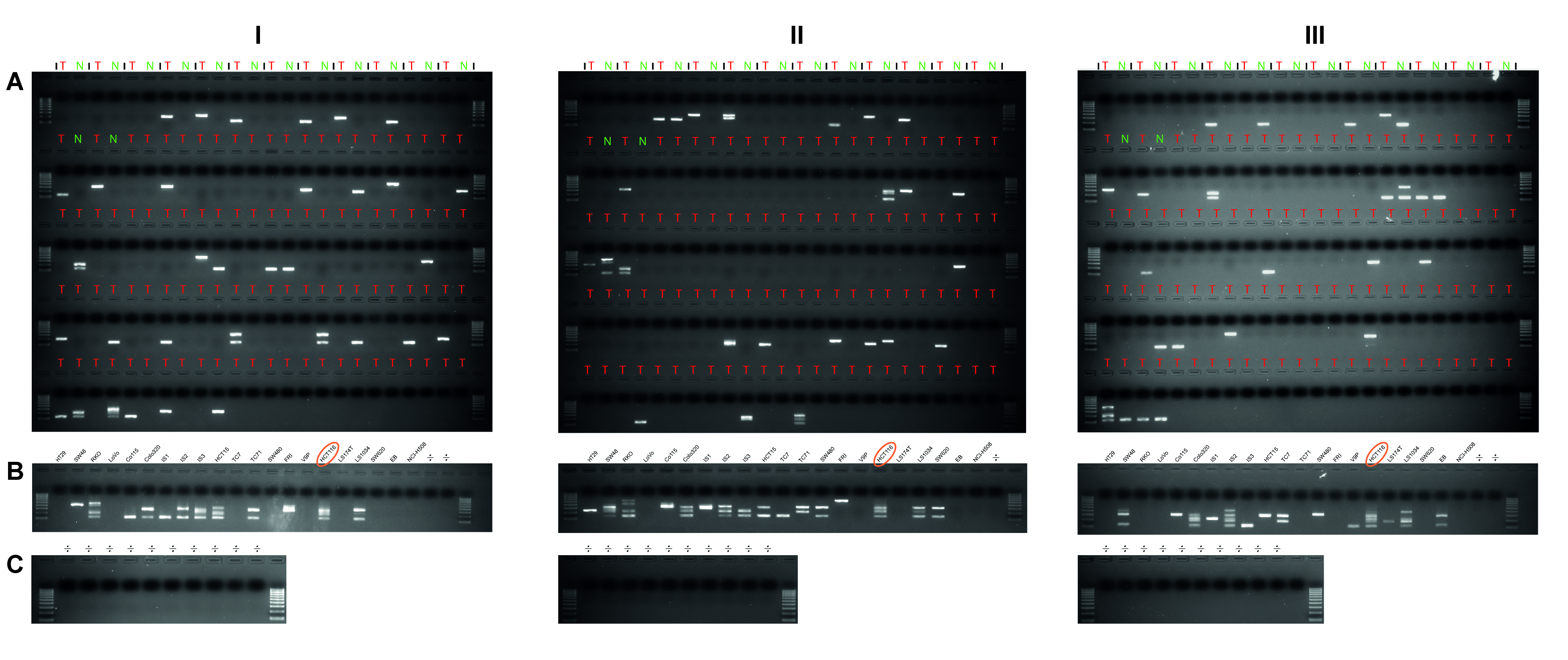

Supplement: Figure S2 — TCF7L2-RP11-57H14.3 : Nested-PCR products in tumor samples, matched normals and CRC cell lines run in triplicate and analyzed on 2% agarose gels. The results diverge somewhat for each replicate. A) Nested-PCR results from patient series 1 and 2. B) Nested-PCR results from the 21 CRC cell lines and some negative controls. There are at least three PCR-products from the cell line HCT116, which are present and identical in all replicates. C) Additional negative controls, including no template controls from cDNA synthesis, first-round and second-round PCR. (TIF) [file pone.0091264.s002.tif]
