# Supplementary material for: High Frequency of Fusion Transcripts Involving TCF7L2 in Colorectal Cancer: Novel Fusion Partner and Splice Variants
Source: PLoS One. 2014 Mar 7;9(3):e91264. doi: 10.1371/journal.pone.0091264 (PMC3946716; doi:10.1371/journal.pone.0091264)
Supplement: Table S1 — Primers used in this study. (DOCX) [file pone.0091264.s003.docx]

**Table S1: Primers used in this study.**

| Primer name: | 5’-3’ primer sequence: | Melting temperature (Tm^a^) |
| --- | --- | --- |
| VTI1A_5’UTR_F (first round) | TTTCCCTGACCTAGGCTTTG | 62°C |
| TCF7L2_Ex6_R (first round) | GGATGGGGGATTTGTCCTAC | 62°C |
| VTI1A_Ex1_F (second round) | CCGACTTCGAAGGTTACGAG | 62°C |
| TCF7L2_ex5_R (second round) | TACGTCGGCTGGTAAGTGTG | 62°C |
| RP11-57H14.3_F1 (first round) | TCCTGGAGATGCCTCTGAGT | 58°C |
| TCF7L2_R1 (first round) | CTACCTCCCCAACGGATCG | 58°C |
| RP11-57H14.3_F2 (second round) | CAAAGCGTGGTCTCATTCCT | 57°C |
| TCF7L2_R2 (second round) | CAGGGAGCCTCCAGAGTAGA | 57°C |
| TCF7L2_DNA_F (genomic breakpoint) | TGGGTGCTGTGCTATGTGTT | 60°C |
| RP11_DNA_R (genomic breakpoint) | GGTAGAGGTTGGCTGCAGTT | 60°C |

a) Melting temperature (Tm) used for optimal primer annealing step during PCR. Annealing temperature was set to the average Tm of the participating primer pair.
