# Supplementary material for: High Frequency of Fusion Transcripts Involving TCF7L2 in Colorectal Cancer: Novel Fusion Partner and Splice Variants
Source: PLoS One. 2014 Mar 7;9(3):e91264. doi: 10.1371/journal.pone.0091264 (PMC3946716; doi:10.1371/journal.pone.0091264)
Supplement: Table S2 — Both fusion transcripts involving TCF7L2 were detected, by RT-PCR, in several human tissues. (DOCX) [file pone.0091264.s004.docx]

**Table S2: Both fusion transcripts involving *TCF7L2* were detected, by RT-PCR, in several human tissues.**

| Tissue | *VTI1A-TCF7L2* | *TCF7L2-RP11-57H14.3* |
| --- | --- | --- |
| Adipose | N | Y |
| Bladder | N | Y |
| Brain | Y | Y |
| Cervix | N | Y |
| Colon | Y | Y |
| Esophagus | N | Y |
| Heart | N | Y |
| Kidney | Y | Y |
| Liver | N | N |
| Lung | Y | Y |
| Ovary | N | Y |
| Placenta | Y | Y |
| Prostate | N | N |
| Skeletal muscle | N | N |
| Spleen | N | N |
| Stomach | N | N |
| Testes | N | Y |
| Thymus | N | Y |
| Thyroid | N | Y |
| Trachea | N | Y |
