# Supplementary material for: High Frequency of Fusion Transcripts Involving TCF7L2 in Colorectal Cancer: Novel Fusion Partner and Splice Variants
Source: PLoS One. 2014 Mar 7;9(3):e91264. doi: 10.1371/journal.pone.0091264 (PMC3946716; doi:10.1371/journal.pone.0091264)
Supplement: Table S3 — Total overview of nested RT-PCR and Sanger sequencing confirmation of fusion transcripts. (DOCX) [file pone.0091264.s005.docx]

**Table S3: Total overview of nested RT-PCR and Sanger sequencing confirmation of fusion transcripts.**

| Sample | Type | MSI-Status | Patient Series | *VTI1A-TCF7L2* #1 | *VTI1A-TCF7L2* #2 | *VTI1A-TCF7L2* #3 | SUM *VTI1A-TCF7L2* Status | Confirmed exon-exon breakpoint *VTI1A-TCF7L2* | *TCF7L2-RP11-57H14.3* #1 | *TCF7L2-RP11-57H14.3* #2 | *TCF7L2-RP11-57H14.3* #3 | SUM *TCF7L2-RP11-57H14.3* | Confirmed exon-exon breakpoint TCF7L2-RP11-57H14.3 |
| --- | --- | --- | --- | --- | --- | --- | --- | --- | --- | --- | --- | --- | --- |
| 012 | tumor | MSI-H | 1 | 1 | 1 | 1 | **3** | EX3(V)-EX6(T) | 0 | 0 | 0 | **0** |  |
| 012_norm | normal |  | 1 | 0 | 0 | 1 | **1** |  | 0 | 0 | 0 | **0** |  |
| 029 | tumor | MSI-L | 1 | 1 | 1 | 0 | **2** | EX3(V)-EX6(T) | 0 | 0 | 0 | **0** |  |
| 029_norm | normal |  | 1 | 0 | 0 | 0 | **0** |  | 0 | 0 | 0 | **0** |  |
| 041 | tumor | MSS | 1 | 1 | 1 | 0 | **2** | EX7(V)-EX4(T)-EX6(T) | 0 | 1 | 0 | **1** | EX4(T)-EX1(RP)-EX3(RP) |
| 041_norm | normal |  | 1 | 0 | 0 | 0 | **0** |  | 0 | 1 | 0 | **1** | EX4(T)-EX1(RP)-EX3(RP) |
| 042 | tumor | MSI-L | 1 | 1 | 0 | 0 | **1** |  | 1 | 1 | 1 | **3** | EX4(T)-EX1(RP)-EX2(RP)-EX3(RP) |
| 042_norm | normal |  | 1 | 0 | 0 | 0 | **0** |  | 0 | 0 | 0 | **0** |  |
| 045 | tumor | MSI-H | 1 | 1 | 0 | 0 | **1** |  | 1 | 1 | 0 | **2** |  |
| 045_norm | normal |  | 1 | 0 | 0 | 0 | **0** |  | 0 | 0 | 1 | **1** |  |
| 065 | tumor | MSS | 1 | 0 | 1 | 0 | **1** | EX7(V)-EX4(T)-EX6(T) | 1 | 0 | 0 | **1** |  |
| 065_norm | normal |  | 1 | 0 | 0 | 0 | **0** |  | 0 | 0 | 0 | **0** |  |
| 067 | tumor | MSS | 1 | NA | 0 | 0 | **0** |  | 0 | 0 | 0 | **0** |  |
| 067_norm | normal |  | 1 | 0 | 0 | 0 | **0** |  | 0 | 0 | 0 | **0** |  |
| 070 | tumor | MSS | 1 | NA | 0 | 0 | **0** |  | 1 | 1 | 1 | **3** | EX4(T)-EX3(RP) |
| 070_norm | normal |  | 1 | 0 | 0 | 0 | **0** |  | 0 | 0 | 0 | **0** |  |
| 072 | tumor | MSI-H | 1 | 1 | 1 | 0 | **2** | EX5(V)-EX6(T) | 1 | 1 | 1 | **3** | EX4(T)-EX1(RP)-EX2(RP)-EX3(RP) |
| 072_norm | normal |  | 1 | 0 | 1 | 1 | **2** | EX5(V)-EX6(T) | 0 | 0 | 1 | **1** |  |
| 074 | tumor | MSS | 1 | 0 | 0 | 0 | **0** |  | 0 | 1 | 0 | **1** | EX4(T)-EX2(RP)-EX3(RP) |
| 074_norm | normal |  | 1 | 1 | 0 | 0 | **1** |  | 1 | 0 | 0 | **1** |  |
| 086 | tumor | MSI-H | 1 | 1 | 1 | 0 | **2** | no* | 0 | 0 | 0 | **0** |  |
| 086_norm | normal |  | 1 | 0 | 0 | 0 | **0** |  | 0 | 0 | 0 | **0** |  |
| 092 | tumor | MSS | 1 | 0 | 1 | 0 | **1** | EX3(V)-EX6(T) | 0 | 0 | 0 | **0** |  |
| 092_norm | normal |  | 1 | 0 | 0 | 0 | **0** |  | 0 | 0 | 0 | **0** |  |
| 096 | tumor | MSS | 1 | 0 | 1 | 0 | **1** | EX5(V)-EX6(T) | 1 | 0 | 1 | **2** |  |
| 096_norm | normal |  | 1 | 0 | 0 | 0 | **0** |  | 0 | 0 | 0 | **0** |  |
| 098 | tumor | MSS | 1 | 1 | 0 | 0 | **1** |  | 1 | 1 | 1 | **3** | EX4(T)-EX1(RP)-EX2(RP)-EX3(RP) |
| 098_norm | normal |  | 1 | 1 | 0 | 0 | **1** |  | 0 | 0 | 0 | **0** |  |
| C1011II | tumor | MSS | 2 | 1 | 1 | 1 | **3** | EX7(V)-EX4(T)-EX6(T) | 0 | 0 | 0 | **0** |  |
| C1022II | tumor | MSI-H | 2 | 1 | 0 | 0 | **1** |  | 0 | 0 | 0 | **0** |  |
| C1030II | tumor | MSI-L | 2 | 0 | 0 | 0 | **0** |  | 0 | 0 | 0 | **0** |  |
| C1033III | tumor | MSS | 2 | 0 | 1 | 0 | **1** | EX1(V)-EX6(T) | 0 | 0 | 0 | **0** |  |
| C1034III | tumor | MSS | 2 | 0 | 1 | 0 | **1** | EX7(V)-EX6(T) | 0 | 0 | 0 | **0** |  |
| C1043II | tumor | MSS | 2 | 1 | 0 | 0 | **1** |  | 1 | 0 | 0 | **1** |  |
| C1049II | tumor | MSS | 2 | 0 | 0 | 1 | **1** |  | 1 | 1 | 0 | **2** | EX4(T)-EX1(RP)-EX3(RP) |
| C1068III | tumor | MSS | 2 | 1 | 0 | 0 | **1** |  | 1 | 0 | 1 | **2** |  |
| C1077III | tumor | MSS | 2 | 1 | 0 | 0 | **1** |  | 0 | 0 | 0 | **0** |  |
| C1079II | tumor | MSS | 2 | 0 | 0 | 0 | **0** |  | 0 | 0 | 0 | **0** |  |
| C1085II | tumor | MSS | 2 | 0 | 0 | 0 | **0** |  | 1 | 0 | 0 | **1** |  |
| C1089II | tumor | MSS | 2 | 0 | 0 | 0 | **0** |  | 0 | 0 | 0 | **0** |  |
| C1091II | tumor | MSI-L | 2 | 1 | 0 | 0 | **1** |  | 1 | 0 | 0 | **1** |  |
| C1102II | tumor | MSS | 2 | 0 | 0 | 0 | **0** |  | 1 | 0 | 0 | **1** |  |
| C1103III | tumor | MSI-L | 2 | 0 | 0 | 0 | **0** |  | 0 | 0 | 0 | **0** |  |
| C1112III | tumor | MSS | 2 | 0 | 0 | 0 | **0** |  | 0 | 0 | 1 | **1** |  |
| C1118III | tumor | MSS | 2 | 0 | 0 | 0 | **0** |  | 0 | 0 | 0 | **0** |  |
| C1122II | tumor | MSS | 2 | 0 | 0 | 0 | **0** |  | 0 | 0 | 0 | **0** |  |
| C1135II | tumor | MSS | 2 | 0 | 0 | 0 | **0** |  | 0 | 0 | 1 | **1** |  |
| C1142III | tumor | MSS | 2 | 0 | 0 | 1 | **1** |  | 0 | 0 | 0 | **0** |  |
| C1144III | tumor | MSS | 2 | 1 | 1 | 0 | **2** | EX3(V)-EX6(T) | 0 | 0 | 0 | **0** |  |
| C1145III | tumor | MSS | 2 | 0 | 0 | 0 | **0** |  | 1 | 1 | 0 | **2** | EX4(T)-EX1(RP)-EX2(RP)-EX3(RP) |
| C1152III | tumor | MSS | 2 | 0 | 0 | 0 | **0** |  | 0 | 0 | 0 | **0** |  |
| C1156II | tumor | MSS | 2 | 0 | 1 | 0 | **1** | EX3(V)-EX6(T) | 0 | 1 | 0 | **1** | EX4(T)-EX1(RP)-EX2(RP)-EX3(RP) |
| C1159III | tumor | MSS | 2 | 0 | 0 | 0 | **0** |  | 0 | 0 | 0 | **0** |  |
| C1165III | tumor | MSS | 2 | 0 | 1 | 0 | **1** | EX5(V)-EX6(T) | 0 | 0 | 0 | **0** |  |
| C1190II | tumor | MSI-H | 2 | 0 | 0 | 0 | **0** |  | 0 | 0 | 0 | **0** |  |
| C1198III | tumor | MSS | 2 | 0 | 0 | 0 | **0** |  | 1 | 0 | 1 | **2** |  |
| C1251III | tumor | MSS | 2 | 1 | 0 | 0 | **1** |  | 1 | 0 | 1 | **2** |  |
| C1257II | tumor | NA | 2 | 0 | 0 | 0 | **0** |  | 0 | 0 | 1 | **1** |  |
| C1263II | tumor | MSS | 2 | 0 | 0 | 0 | **0** |  | 1 | 0 | 0 | **1** |  |
| C1264II | tumor | MSS | 2 | 0 | 0 | 0 | **0** |  | 1 | 1 | 1 | **3** | EX4(T)-EX3(RP) |
| C1267III | tumor | MSS | 2 | 0 | 0 | 0 | **0** |  | 1 | 0 | 0 | **1** |  |
| C1271III | tumor | MSS | 2 | 0 | 0 | 0 | **0** |  | 0 | 0 | 0 | **0** |  |
| C1273II | tumor | MSI-H | 2 | 0 | 0 | 0 | **0** |  | 0 | 0 | 0 | **0** |  |
| C1275III | tumor | MSS | 2 | 0 | 0 | 0 | **0** |  | 1 | 0 | 0 | **1** |  |
| C1280III | tumor | MSS | 2 | 0 | 0 | 0 | **0** |  | 0 | 0 | 0 | **0** |  |
| C1283II | tumor | MSI-H | 2 | 1 | 0 | 0 | **1** |  | 1 | 0 | 1 | **2** |  |
| C1284III | tumor | MSS | 2 | 1 | 1 | 1 | **3** | EX7(V)-EX6(T) | 0 | 0 | 0 | **0** |  |
| C1285III | tumor | MSS | 2 | 0 | 0 | 0 | **0** |  | 0 | 0 | 1 | **1** |  |
| C1286III | tumor | MSS | 2 | 0 | 0 | 0 | **0** |  | 0 | 0 | 0 | **0** |  |
| C1291II | tumor | MSS | 2 | 0 | 0 | 0 | **0** |  | 0 | 0 | 0 | **0** |  |
| C1292III | tumor | MSS | 2 | 0 | 0 | 0 | **0** |  | 1 | 1 | 0 | **2** |  |
| C1294II | tumor | MSS | 2 | 0 | 0 | 0 | **0** |  | 0 | 0 | 0 | **0** |  |
| C1296II | tumor | MSS | 2 | 0 | 0 | 0 | **0** |  | 1 | 0 | 0 | **1** |  |
| C1301III | tumor | MSS | 2 | 0 | 0 | 0 | **0** |  | 0 | 0 | 0 | **0** |  |
| C1314III | tumor | MSI-H | 2 | 0 | 0 | 0 | **0** |  | 0 | 1 | 0 | **1** |  |
| C1321II | tumor | MSS | 2 | 0 | 0 | 0 | **0** |  | 0 | 0 | 0 | **0** |  |
| C1323III | tumor | MSS | 2 | 1 | 0 | 0 | **1** |  | 0 | 0 | 0 | **0** |  |
| C1330III | tumor | MSS | 2 | 0 | 0 | 0 | **0** |  | 0 | 0 | 0 | **0** |  |
| C1333III | tumor | MSS | 2 | 1 | 0 | 0 | **1** |  | 0 | 0 | 0 | **0** |  |
| C1334III | tumor | MSS | 2 | 0 | 0 | 0 | **0** |  | 0 | 0 | 1 | **1** |  |
| C1338III | tumor | MSS | 2 | 0 | 0 | 0 | **0** |  | 0 | 0 | 0 | **0** |  |
| C1340III | tumor | MSI-L | 2 | 0 | 0 | 0 | **0** |  | 0 | 1 | 0 | **1** | EX4(T)-EX2(RP)-EX3(RP) |
| C1350II | tumor | MSS | 2 | 0 | 0 | 0 | **0** |  | 0 | 0 | 0 | **0** |  |
| C1355III | tumor | MSS | 2 | 0 | 0 | 0 | **0** |  | 0 | 0 | 0 | **0** |  |
| C1356III | tumor | MSS | 2 | 0 | 0 | 0 | **0** |  | 0 | 0 | 0 | **0** |  |
| C1357II | tumor | MSI-L | 2 | 0 | 0 | 1 | **1** |  | 0 | 1 | 0 | **1** |  |
| C1364II | tumor | MSS | 2 | 0 | 0 | 0 | **0** |  | 0 | 0 | 0 | **0** |  |
| C1379II | tumor | MSS | 2 | 0 | 0 | 1 | **1** |  | 0 | 0 | 0 | **0** |  |
| C1380III | tumor | MSS | 2 | 0 | 0 | 0 | **0** |  | 0 | 0 | 0 | **0** |  |
| C1389III | tumor | MSS | 2 | 0 | 0 | 0 | **0** |  | 1 | 1 | 0 | **2** | EX4(T)-EX1(RP)-EX3(RP) |
| C1391II | tumor | MSS | 2 | 0 | 0 | 0 | **0** |  | 0 | 0 | 0 | **0** |  |
| C1393III | tumor | MSS | 2 | 1 | 0 | 0 | **1** |  | 0 | 0 | 0 | **0** |  |
| C1395II | tumor | MSS | 2 | 0 | 0 | 1 | **1** |  | 1 | 1 | 0 | **2** | EX4(T)-EX1(RP)-EX2(RP)-EX3(RP) |
| C1402III | tumor | MSS | 2 | 0 | 0 | 0 | **0** |  | 0 | 0 | 0 | **0** |  |
| C844II | tumor | MSS | 2 | 0 | 0 | 0 | **0** |  | 0 | 0 | 0 | **0** |  |
| C861y | tumor | MSS | 2 | 0 | 0 | 1 | **1** |  | 0 | 0 | 0 | **0** |  |
| C874III | tumor | MSS | 2 | 1 | 1 | 0 | **2** | EX3(V)-EX4(T)-EX6(T) | 0 | 0 | 0 | **0** |  |
| C891II | tumor | MSI-L | 2 | 1 | 0 | 0 | **1** |  | 1 | 0 | 1 | **2** |  |
| C895II | tumor | MSS | 2 | 1 | 0 | 0 | **1** |  | 0 | 0 | 0 | **0** |  |
| C896III | tumor | MSS | 2 | 0 | 0 | 0 | **0** |  | 0 | 0 | 0 | **0** |  |
| C903II | tumor | MSS | 2 | 0 | 0 | 0 | **0** |  | 0 | 0 | 0 | **0** |  |
| C914III | tumor | MSS | 2 | 0 | 0 | 0 | **0** |  | 0 | 0 | 0 | **0** |  |
| C932III | tumor | MSS | 2 | 0 | 0 | 0 | **0** |  | 0 | 0 | 0 | **0** |  |
| C935II | tumor | MSS | 2 | 0 | 0 | 0 | **0** |  | 0 | 0 | 0 | **0** |  |
| C937III | tumor | MSS | 2 | 0 | 0 | 0 | **0** |  | 0 | 0 | 0 | **0** |  |
| C938III | tumor | MSS | 2 | 0 | 0 | 0 | **0** |  | 1 | 0 | 0 | **1** |  |
| C940III | tumor | MSS | 2 | 1 | 0 | 0 | **1** |  | 1 | 0 | 1 | **2** |  |
| C950II | tumor | MSS | 2 | 1 | 0 | 1 | **2** |  | 0 | 0 | 0 | **0** |  |
| C963III | tumor | MSS | 2 | 1 | 0 | 0 | **1** |  | 0 | 0 | 1 | **1** |  |
| C964II | tumor | MSS | 2 | 0 | 1 | 0 | **1** | EX1(V)-EX6(T) | 1 | 1 | 1 | **3** |  |
| C965II | tumor | MSI-H | 2 | 0 | 0 | 0 | **0** |  | 0 | 1 | 1 | **2** | EX4(T)-EX1(RP)-EX2(RP)-EX3(RP) |
| C970II | tumor | MSS | 2 | 0 | 0 | 0 | **0** |  | 1 | 0 | 1 | **2** |  |
| C975III | tumor | MSS | 2 | 0 | 0 | 1 | **1** |  | 0 | 0 | 0 | **0** |  |
| C976II | tumor | MSS | 2 | 0 | 0 | 0 | **0** |  | 0 | 1 | 0 | **1** | EX4(T)-EX2(RP)-EX3(RP) |
| C978II | tumor | MSS | 2 | 0 | 1 | 0 | **1** |  | 0 | 0 | 0 | **0** |  |
| C980II | tumor | MSI-H | 2 | 0 | 0 | 0 | **0** |  | 1 | 0 | 0 | **1** |  |
| C981III | tumor | MSS | 2 | 0 | 0 | 0 | **0** |  | 0 | 0 | 0 | **0** |  |
| C982III | tumor | MSI-L | 2 | 0 | 0 | 0 | **0** |  | 0 | 1 | 0 | **1** | EX4(T)-EX1(RP)-EX2(RP)-EX3(RP) |
| C983III | tumor | MSS | 2 | 0 | 1 | 1 | **2** | EX7(V)-EX4(T)-EX6(T) | 1 | 1 | 0 | **2** |  |
| C985III | tumor | MSS | 2 | 0 | 1 | 1 | **2** |  | 0 | 0 | 1 | **1** |  |
| Co115 | Cell line |  |  | 0 | 0 | 0 | **0** |  | 1 | 1 | 1 | **3** |  |
| Colo320 | Cell line |  |  | 1 | 1 | 0 | **2** | EX5(V)-EX6(T) | 1 | 1 | 1 | **3** |  |
| EB | Cell line |  |  | 0 | 1 | 1 | **2** | EX7(V)-EX6(T) | 0 | 0 | 1 | **1** |  |
| FRI | Cell line |  |  | 1 | 1 | 0 | **2** | EX7(V) EX4(T)-EX6(T) | 1 | 1 | 0 | **2** | EX4(T)-EX5,6,7(V)-EX1,2,3(RP) |
| HCT116 | Cell line |  |  | 1 | 0 | 1 | **2** |  | 1 | 1 | 1 | **3** | Several† |
| HCT15 | Cell line |  |  | 0 | 0 | 0 | **0** |  | 1 | 1 | 1 | **3** |  |
| HT29 | Cell line |  |  | 0 | 0 | 0 | **0** |  | 0 | 1 | 0 | **1** | EX4(T)-EX2(RP)-EX3(RP) |
| IS1 | Cell line |  |  | 1 | 0 | 1 | **2** |  | 1 | 1 | 1 | **3** |  |
| IS2 | Cell line |  |  | 1 | 0 | 1 | **2** |  | 1 | 1 | 1 | **3** |  |
| IS3 | Cell line |  |  | 0 | 0 | 1 | **1** |  | 1 | 1 | 1 | **3** |  |
| LoVo | Cell line |  |  | 0 | 0 | 0 | **0** |  | 0 | 0 | 0 | **0** |  |
| LS1034 | Cell line |  |  | 0 | 0 | 0 | **0** |  | 1 | 1 | 1 | **3** |  |
| LS174T | Cell line |  |  | 0 | 0 | 0 | **0** |  | 0 | 0 | 1 | **1** |  |
| NCIH508 | Cell line |  |  | 1 | 1 | 1 | **3** | EX2(V)-EX6(T) | 0 | 0 | 0 | **0** |  |
| RKO | Cell line |  |  | 0 | 0 | 0 | **0** |  | 0 | 1 | 1 | **2** |  |
| SW48 | Cell line |  |  | 0 | 0 | 0 | **0** |  | 1 | 1 | 1 | **3** |  |
| SW480 | Cell line |  |  | 1 | 0 | 1 | **2** |  | 1 | 1 | 0 | **2** |  |
| SW620 | Cell line |  |  | 0 | 1 | 0 | **1** | EX1(V)-EX6(T) | 0 | 1 | 0 | **1** |  |
| TC7 | Cell line |  |  | 0 | 0 | 0 | **0** |  | 0 | 1 | 1 | **2** | EX4(T)-EX3(RP) |
| TC71 | Cell line |  |  | 0 | 1 | 0 | **1** | EX3(V)-EX4(T)-EX6(T) | 1 | 1 | 0 | **2** |  |
| V9P | Cell line |  |  | 0 | 0 | 0 | **0** |  | 0 | 0 | 1 | **1** |  |
| Adipose | Normal tissue |  |  | 0 | 0 | 0 | **0** |  | 1 | 0 | 1 | **2** |  |
| Bladder | Normal tissue |  |  | 0 | 0 | 0 | **0** |  | 1 | 1 | 0 | **2** |  |
| Brain | Normal tissue |  |  | 0 | 0 | 1 | **1** |  | 1 | 1 | 1 | **3** |  |
| Cervix | Normal tissue |  |  | 0 | 0 | 0 | **0** |  | 1 | 1 | 0 | **2** | EX4(T)-EX1(RP)-EX2(RP)-EX3(RP) |
| Colon | Normal tissue |  |  | 0 | 0 | 1 | **1** |  | 1 | 1 | 1 | **3** |  |
| Esophagus | Normal tissue |  |  | 0 | 0 | 0 | **0** |  | 0 | 1 | 0 | **1** | EX4(T)-EX1(RP)-EX2(RP)-EX3(RP) |
| Heart | Normal tissue |  |  | 0 | 0 | 0 | **0** |  | 1 | 1 | 0 | **2** | EX4(T)-EX1(RP)-EX3(RP) |
| Kidney | Normal tissue |  |  | 1 | 1 | 0 | **2** |  | 1 | 1 | 1 | **3** |  |
| Liver | Normal tissue |  |  | 0 | 0 | 0 | **0** |  | 0 | 0 | 0 | **0** |  |
| Lung | Normal tissue |  |  | 1 | 1 | 0 | **2** |  | 1 | 0 | 1 | **2** |  |
| Ovary | Normal tissue |  |  | 0 | 0 | 0 | **0** |  | 1 | 1 | 0 | **2** | EX4(T)-EX3(RP) |
| Placenta | Normal tissue |  |  | 0 | 1 | 0 | **1** |  | 1 | 1 | 0 | **2** | EX4(T)-EX2(RP)-EX3(RP) |
| Prostate | Normal tissue |  |  | 0 | 0 | 0 | **0** |  | 0 | 0 | 0 | **0** |  |
| Skeletal_muscle | Normal tissue |  |  | 0 | 0 | 0 | **0** |  | 0 | 0 | 0 | **0** |  |
| Spleen | Normal tissue |  |  | 0 | 0 | 0 | **0** |  | 0 | 0 | 0 | **0** |  |
| Stomach | Normal tissue |  |  | 0 | 0 | 0 | **0** |  | 0 | 0 | 0 | **0** |  |
| Testes | Normal tissue |  |  | 0 | 0 | 0 | **0** |  | 1 | 1 | 1 | **3** | EX4(T)-EX3(RP) |
| Thymus | Normal tissue |  |  | 0 | 0 | 0 | **0** |  | 1 | 0 | 0 | **1** |  |
| Thyroid | Normal tissue |  |  | 0 | 0 | 0 | **0** |  | 1 | 1 | 1 | **3** | EX4(T)-EX1(RP)-EX2(RP)-EX3(RP) |
| Trachea | Normal tissue |  |  | 0 | 0 | 0 | **0** |  | 1 | 0 | 0 | **1** |  |

* The nested-PCR product from tumor sample 086 (from series 1) was Sanger sequenced. However, no clear exon-exon breakpoint was found, as the sequence spanned from one codon on exon 7 of *VTI1A* to the middle of exon 6 of *TCF7L2*.

† Based on gel band lengths, all four fusion transcript variants of *TCF7L2-RP11-57H14.3* were expressed in the HCT116 cell line.
